# CARE-AD: a multi-agent large language model framework for Alzheimer’s disease prediction using longitudinal clinical notes

**DOI:** 10.1038/s41746-025-01940-4

**Published:** 2025-08-24

**Authors:** Rumeng Li, Xun Wang, Dan Berlowitz, Jesse Mez, Honghuang Lin, Hong Yu

**Affiliations:** 1 Manning College of Information & Computer Sciences, University of Massachusetts Amherst, Amherst, MA USA; 2 Center for Health Organization & Implementation Research, VA Bedford Health Care System, Bedford, MA USA; 3 Microsoft Corporation, Redmond, WA USA; 4 Department of Public Health, University of Massachusetts Lowell, Lowell, MA USA; 5 Center of Biomedical and Health Research in Data Sciences, University of Massachusetts Lowell, Lowell, MA USA; 6 Chobanian & Avedisian School of Medicine, Boston University, Boston, MA USA; 7 Department of Medicine, UMass Chan Medical School, Worcester, MA USA; 8 Miner School of Computer and Information Sciences, University of Massachusetts Lowell, Lowell, MA USA

**Keywords:** Cognitive ageing, Alzheimer's disease, Machine learning, Predictive medicine

## Abstract

Large language models (LLMs) have shown promising capabilities across diverse domains, yet their application to complex clinical prediction tasks remains limited. In this study, we present CARE-AD (Collaborative Analysis and Risk Evaluation for Alzheimer’s Disease), a multi-agent LLM-based framework for forecasting Alzheimer’s disease (AD) onset by analyzing longitudinal electronic health record (EHR) notes. CARE-AD assigns specialized LLM agents to extract signs and symptoms relevant to AD and conduct domain-specific evaluations—emulating a collaborative diagnostic process. In a retrospective evaluation, CARE-AD achieved higher accuracy (0.53 vs. 0.26–0.45) than baseline single-model approaches in predicting AD risk 10 years prior to the first recorded diagnosis code. These findings highlight the feasibility of using multi-agent LLM systems to support early risk assessment for AD and motivate further research on their integration into clinical decision support workflows.

## Introduction

Alzheimer’s disease (AD) is a progressive neurodegenerative disorder characterized by cognitive decline, memory impairment, and functional deterioration, ultimately leading to loss of independence in affected individuals^1^. Being the most common cause of dementia worldwide, AD imposes a significant burden on patients, caregivers, and healthcare systems^2^. With the aging global population, the prevalence of AD is expected to rise substantially in the coming decades, underscoring the urgent need for early detection and effective management strategies^2^.

Although formal diagnosis of AD typically involves cognitive assessments and biomarker-based tests, these procedures are often costly, invasive, and impractical for large-scale screening and expensive, limiting their widespread adoption in clinical practice^3–5^. Meanwhile, studies have identified early indicators of AD risk that emerge well before formal diagnosis^6^. Subjective cognitive decline and prodromal symptoms of AD dementia frequently manifest years in advance, involving subtle and often neglected changes in memory, cognition, and behavior^6–9^. Recognizing these indicators is crucial for early AD prediction and intervention^10^. Nevertheless, such signs are often overlooked because they are frequently described within unstructured electronic health record (EHR) notes rather than documented in standardized fields such as International Classification of Diseases (ICD) codes or lab results^2,11^. As a result, much of the critical pre-diagnostic information remains underutilized.

Previous research has explored the use of structured EHR data for early AD prediction^12–19^, but relatively few studies have incorporated unstructured narratives. Existing NLP efforts have largely focused on isolated symptoms or specific note types, limiting their generalizability across longitudinal clinical records^11,20–27^. Recent advances in large language models (LLMs), such as OpenAI’s GPT-4 and Meta’s LLaMA family, offer new opportunities to extract complex patterns from free-text data^28–30^. However, significant challenges remain for healthcare applications, including data privacy, model scalability, and the limitations of single-model reasoning in capturing the multidimensional nature of clinical decision-making^31^.

To address these challenges, we drew inspiration from the clinical diagnostic process for AD, which relies on a rigorous multidisciplinary approach. In clinical practice, specialists in neurology, psychiatry, geriatrics, primary care, and other relevant fields, each contribute complementary expertise to comprehensively assess patient risk^32–34^. This collaborative model is essential for evaluating multifactorial conditions like AD, where diverse symptom domains must be integrated for an accurate and nuanced assessment.

We propose to simulate this clinical procedure through a multi-agent framework, with each agent representing a specialist domain. By mimicking the collaboration of clinicians, this design aims to enhance predictive performance and interpretability. Multi-agent methods have shown promise in healthcare tasks such as medical question answering^35^ and mitigating cognitive biases in clinical decision-making^36^. Coordinating specialized agents not only improves prediction accuracy but also yields clearer intermediate reasoning steps—an important factor for clinical transparency and trust. This approach is also conceptually aligned with the Mixture of Experts (MoE) paradigm^37^, which demonstrates that specialization across expert components can improve performance on complex tasks.

Building on these insights from clinical practice and model specialization, we developed CARE-AD (Collaborative Analysis & Risk Evaluation for Alzheimer’s Disease)—a multi-agent LLM framework designed to predict AD risk from longitudinal unstructured EHR data. CARE-AD simulates a virtual multidisciplinary consultation: agents representing clinical domains such as primary care, neurology, psychiatry, geriatrics, and psychology analyze a patient’s symptom trajectory and provide domain-specific assessments. These are then synthesized by an AD specialist agent into an individualized risk prediction. By modeling temporal symptom patterns and incorporating diverse clinical perspectives, CARE-AD aims to improve sensitivity to early AD-related signs—especially those often underrepresented in structured records—while enhancing interpretability through agent-specific contributions that clinicians can review.

While further validation in real-world clinical workflows is needed, this study presents the design and evaluation of CARE-AD on a large dataset from the U.S. Veterans Health Administration (VHA), demonstrating its potential to improve early AD risk stratification and support more informed clinical decision-making.

## Results

**Fig. 1:**
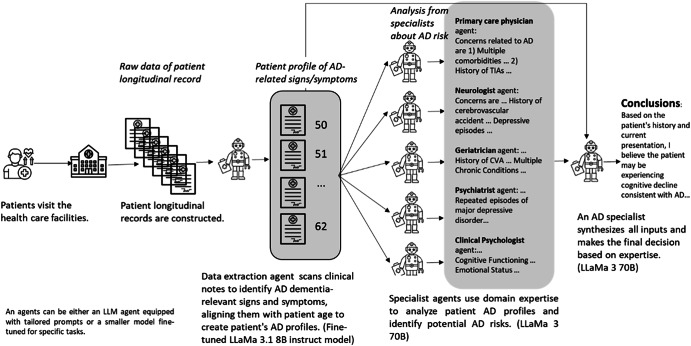
Illustration of an example patient processed by the CARE-AD framework for early AD prediction using longitudinal EHRs.

The CARE-AD prediction framework involves three steps to assess AD risk. First, a data extraction agent identifies AD-related signs and symptoms from EHR notes organizing them into age-aware patient profiles categorized by specific symptom types. Second, a multidisciplinary team of specialist agents—including a primary care physician agent for holistic assessment, neurologist and psychiatrist agents for neurological and psychiatric evaluation, a geriatrician agent for assessing daily living and independence, and a clinical psychologist agent for behavioral and psychological analysis—conducts a coordinated, domain-specific evaluation. Finally, an AD-focused specialist agent synthesizes these insights to generate a robust AD risk assessment. The framework is illustrated in Fig. 1. A detailed description of the system overview and technical architecture is provided in Supplementary Note 1.

### Study sample

Our cohort consists of 17,488 AD cases and 64,691 controls from the VHA. Supplementary Fig. 1 illustrates the cohort creation process. We assessed the CARE-AD prediction framework using a randomly sampled evaluation set of 1000 AD cases and 3627 controls. Demographic details of the evaluation set are presented in Table 1.

**Table 1:** Demographics of the evaluation set

Characteristic	AD case	Control
Patients, No.	1000	3627
AD onset/index time age (SD)	79.0 (8.4)	78.6 (8.6)
Sex, *n* (%)		
Female	18 (1.8%)	57 (1.6%)
Male	982 (98.2%)	3570 (98.4%)
Race no. (%)		
White	793 (79%)	2913 (80.3%)
Black or African American	131 (13.1%)	400 (11.0%)
Other	76 (7.6%)	314 (8.7%)
Ethnicity no. (%)		
Hispanic/Latino	66 (6.6%)	253 (7.0%)
Non-Hispanic/Latino	903 (90.3%)	3282 (90.5%)
Others/Unknown	31 (3.1%)	92 (2.5%)
AD-relevant sentences per year (average)	99	60

### Performance of data extraction agent

Our data extraction agent is designed to identify signs and symptoms of AD from longitudinal EHR notes using a two-step classification process. First, we perform a binary classification to determine whether a candidate sentence contains any AD-related signs or symptoms. Second, for those sentences deemed relevant, we apply a multi-class classification to assign each instance to one of the five expert-defined AD categories: cognitive impairment, notice/concern by others, requiring assistance/functional impairment, physiological changes, and neuropsychiatric symptoms. We trained separate LLaMA 3.1 8B models for each classification step on a dataset derived from previous work (Supplementary Table 1)^38^. Unlike earlier approaches, we excluded categories involving cognitive assessments or diagnostic tests, because our method relies strictly on symptom-based evidence rather than formal clinical investigations of AD. Comprehensive descriptions of each category are available in the Methods section and in Supplementary Note 2.

Table 2 presents the performance of our first fine-tuned LLaMA 3.1 8B model on the binary classification step, demonstrating whether sentences indicate AD-related signs or symptoms. We compare this model against a strong ensemble baseline^38^, which integrates three pretrained language models—BERT (bert-base-uncased), RoBERTa (roberta-base), and ClinicalBERT—fine-tuned on our dataset. Our LLaMA 3.1 8B model outperforms this ensemble model, highlighting its effectiveness on the initial binary decision. Sentences identified as relevant are then processed by our second fine-tuned LLaMA 3.1 8B model, which performs multi-class classification to assign each instance to one of five symptom categories. These category-specific outputs help generate detailed inputs for subsequent LLM agents. The classifier’s evaluation results are reported in Table 3.

**Table 2:** Data extraction agent performance as a binary classifier

	Precision (Positive)	Recall (Positive)	F-1(Positive)	Overall accuracy
Fine-tuned LLaMa 3.1 8B instruct	0.74 (0.72, 0.76)	0.89 (0.88, 0.91)	0.81 (0.79, 0.83)	0.93 (0.91, 0.95)
Ensemble (Li et al. 2023)	0.71 (0.69, 0.73)	0.84 (0.82, 0.86)	0.77 (0.75, 0.79)	0.91 (0.89, 0.93)

**Table 3:** Data extraction agent performance as a multi-class classifier

Symptom category	Precision	Recall	F1-score
Cognitive impairment	0.77 (0.75, 0.78)	0.82 (0.83, 0.84)	0.79 (0.77, 0.81)
Notice/Concern by others	0.84 (0.83, 0.84)	0.39 (0.37, 0.41)	0.53 (0.52, 0.55)
Requires assistance	0.69 (0.67, 0.70)	0.65 (0.63, 0.67)	0.67 (0.65, 0.68)
Physiological changes	0.74 (0.73, 0.75)	0.76 (0.74, 0.77)	0.75 (0.73, 0.77)
Neuropsychiatric symptoms	0.78 (0.77, 0.80)	0.83 (0.81, 0.85)	0.8 (0.78, 0.82)
Overall accuracy	Micro-average	0.75 (0.73, 0.77)	
	Macro-average	0.76 (0.74, 0.78)	

Based on the classified sentences, we constructed longitudinal, AD-specific patient profiles by mapping identified signs and symptoms from EHR notes chronologically to symptom categories and the patient’s age, forming a time series of disease-relevant manifestations. Supplementary Note 3 details the construction process, and Supplementary Note 4 provides an example of an aggregated patient profile. This example illustrates how diverse AD-related symptoms—such as cognitive impairments and physiological changes—are captured and tracked across the patient’s clinical history.

### Multi-agent risk prediction across time points

We evaluated our methodology by predicting AD risk at seven distinct time points: 1 day, 1 year, 2 years, 3 years, 5 years, 7 years, and 10 years prior to the formal ICD-based diagnosis. A multidisciplinary team of specialist agents—including a primary care physician, neurologist, psychiatrist, geriatrician, clinical psychologist, and an AD specialist—collaboratively analyzed patients’ AD profiles within a specific observation window generated by the data extraction agent. Detailed setup and prompts for the agents are provided in Supplementary Note 5. As shown in Table 4, our multi-agent system demonstrated consistent performance across all time points, with an accuracy of 0.83 at −1 day and 0.53 at −10 years. These results suggest the model’s potential to identify both near-term and earlier indicators of AD risk based on longitudinal clinical narratives.

**Table 4:** AD prediction performance of our proposed CARE-AD approach

Prediction Y ear	AD cases			Controls			Accuracy
	Precision	Recall	F1 score	Precision	Recall	F1 score	
−1 day	0.59 (0.57, 0.61)	0.73 (0.70, 0.76)	0.65 (0.63, 0.67)	0.92 (0.91, 0.93)	0.86 (0.85, 0.87)	0.89 (0.88, 0.90)	0.83 (0.82, 0.84)
−1 year	0.47 (0.45, 0.49)	0.66 (0.63, 0.69)	0.55 (0.53, 0.57)	0.89 (0.89, 0.90)	0.79 (0.78, 0.80)	0.84 (0.83, 0.85)	0.76 (0.75, 0.78)
−2 year	0.39 (0.37, 0.41)	0.59 (0.56, 0.62)	0.47 (0.45, 0.49)	0.87 (0.86, 0.88)	0.75 (0.73, 0.76)	0.80 (0.79, 0.81)	0.71 (0.70, 0.73)
−3 year	0.34 (0.32, 0.35)	0.54 (0.51, 0.57)	0.41 (0.39, 0.43)	0.85 (0.84, 0.87)	0.71 (0.69, 0.72)	0.77 (0.76, 0.79)	0.67 (0.66, 0.68)
−5 year	0.26 (0.25, 0.28)	0.46 (0.43, 0.49)	0.33 (0.31, 0.35)	0.81 (0.80, 0.82)	0.65 (0.64, 0.67)	0.72 (0.71, 0.73)	0.61 (0.59, 0.62)
−7 year	0.24 (0.23, 0.26)	0.43 (0.40, 0.46)	0.31 (0.29, 0.33)	0.80 (0.79, 0.81)	0.62 (0.61, 0.64)	0.70 (0.69, 0.71)	0.58 (0.57, 0.60)
−10 year	0.20 (0.18, 0.21)	0.38 (0.35, 0.41)	0.26 (0.24, 0.28)	0.77 (0.76, 0.78)	0.57 (0.55, 0.59)	0.65 (0.64, 0.67)	0.53 (0.51, 0.54)

### Comparison with single-model baselines

For comparison, we also evaluated four baseline methods, each using the same LLaMA 3 70B model: (1) a zero-shot approach with a single LLM call; (2) a Chain of Thought (CoT) approach^39^ that guides language models to reason step by step by generating intermediate reasoning steps before producing a final answer; (3) a self-consistency approach^40^ that generates multiple responses and selects the most consistent output through majority voting; and (4) a self-refine approach^41^ that iteratively revises its outputs to improve clarity and correctness. As shown in Table 5, with an equal number of LLM calls (six), our CARE-AD method consistently outperformed these baselines, demonstrating the benefits of collaborative, domain-specialized reasoning.

**Table 5:** Performance comparison of the proposed CARE-AD method with baseline models at -10-year prediction

Method	LLM calls	AD cases (P/R/F)	Controls (P/R/F)	Accuracy
Zero-shot	1	0.09 (0.08, 0.09)/0.25 (0.23, 0.28)/0.13 (0.11, 0.14)	0.56 (0.54, 0.57)/0.26 (0.25, 0.27)/0.35 (0.34, 0.37)	0.26 (0.25, 0.27)
Chain of thought (CoT)	1	0.11 (0.10, 0.12)/0.27 (0.25, 0.29)/0.15 (0.13, 0.17)	0.65 (0.63, 0.67)/0.37 (0.35, 0.39)/0.47 (0.45, 0.49)	0.35 (0.33, 0.37)
Self-consistency	6 reasoning paths	0.13 (0.11, 0.15)/0.29 (0.26, 0.32)/0.18 (0.16, 0.20)	0.70 (0.69, 0.71)/0.47 (0.45, 0.49)/0.56 (0.54, 0.58)	0.43 (0.42, 0.44)
Self-refine	6 refine rounds	0.16 (0.14, 0.18)/0.36 (0.33, 0.39)/0.22 (0.19, 0.25)	0.73 (0.72, 0.74)/0.47 (0.45, 0.49)/0.57 (0.55, 0.59)	0.45 (0.44, 0.46)
AutoGen multi-agent (1 round)	6 doctor agents (6 LLM calls)	0.16 (0.15, 0.17)/0.36 (0.33, 0.39)/0.22 (0.20, 0.24)	0.73 (0.72, 0.74)/0.48 (0.47, 0.50)/0.58 (0.57, 0.59)	0.45 (0.44, 0.47)
AutoGen multi-agent (2 rounds)	6 doctor agents (12 LLM calls)	0.20 (0.18, 0.21)/0.38 (0.35, 0.41)/0.26 (0.23, 0.28)	0.77 (0.76, 0.78)/0.58 (0.56, 0.59)/0.66 (0.65, 0.67)	0.53 (0.52, 0.55)
AutoGen multi-agent (3 rounds)	6 doctor agents (18 LLM calls)	0.20 (0.18, 0.21)/0.38 (0.35, 0.41)/0.26 (0.24, 0.28)	0.77 (0.76, 0.78)/0.58 (0.56, 0.59)/0.66 (0.64, 0.67)	0.53 (0.52, 0.55)
CARE-AD	6 doctor agents	0.20 (0.18, 0.21)/0.38 (0.35, 0.41)/0.26 (0.24, 0.28)	0.77 (0.76, 0.78)/0.57 (0.55, 0.59)/0.65 (0.64, 0.67)	0.53 (0.51, 0.54)

### Multi-agent conversation baseline

To further strengthen the comparison, we implemented a multi-agent conversational baseline using the AutoGen framework^42^. This setup mirrors the structure of CARE-AD, in which a supervisor agent (AD specialist) engages in multi-round dialogue with five domain-specific doctor agents. As shown in Table 5, the AutoGen-based configuration achieved comparable performance to CARE-AD when using 12 or more LLM calls, but required greater computational cost to match the performance of our more efficient prompt-based design.

### Ablation study

In reviewing these ablation results for prediction at 10 years prior, CARE-AD (the full multi-agent configuration) achieved the highest overall accuracy (0.53). As shown in Table 6, the zero-shot baseline (no agent roles) performed poorly, particularly in identifying AD cases (F-score of 0.13), resulting in the lowest accuracy (0.26). When each specialty doctor agent was individually excluded, performance dropped below that of the full CARE-AD model, indicating that all agent roles contributed positively to classification. Notably, removing the neurologist role reduced accuracy to 0.50, suggesting that neurologist expertise is especially informative for distinguishing AD symptoms. Similarly, excluding the psychiatrist role lowered accuracy to 0.49, underscoring the importance of psychiatric insights in detecting mental health disorders associated with AD. Removing other roles (clinical psychologist, primary care physician, or geriatrician) also resulted in performance declines, though these decreases were comparatively smaller. Overall, the results in Table 6 highlight the value of incorporating multiple complementary clinical perspectives to improve AD vs. control classification accuracy.

**Table 6:** Ablation study showing the impact of agent roles on AD prediction at -10-year prediction

Agents	AD cases (P/R/F)	Controls (P/R/F)	Accuracy
CARE-AD	0.20 (0.18, 0.21)/0.38 (0.35, 0.41)/0.26 (0.24, 0.28)	0.77 (0.76, 0.78)/0.57 (0.55, 0.59)/0.65 (0.64, 0.67)	0.53 (0.51, 0.54)
Zero-shot (No agents)	0.09 (0.08, 0.09)/0.25 (0.23, 0.28)/0.13 (0.11, 0.14)	0.56 (0.54, 0.57)/0.26 (0.25, 0.27)/0.35 (0.34, 0.37)	0.26 (0.25, 0.27)
Exclude neurologist	0.17 (0.16, 0.18)/0.33 (0.30, 0.36)/0.23 (0.21, 0.24)	0.75 (0.74, 0.76)/0.55 (0.53, 0.57)/0.64 (0.62, 0.65)	0.50 (0.49, 0.52)
Exclude clinical psychologist	0.19 (0.17, 0.20)/0.36 (0.33, 0.39)/0.25 (0.23, 0.27)	0.76 (0.75, 0.77)/0.57 (0.55, 0.59)/0.65 (0.64, 0.67)	0.52 (0.51, 0.54)
Exclude psychiatrist	0.17 (0.15, 0.18)/0.34 (0.31, 0.37)/0.23 (0.21, 0.24)	0.75 (0.73, 0.75)/0.53 (0.51, 0.55)/0.62 (0.61, 0.63)	0.49 (0.47, 0.50)
Exclude primary care physician	0.18 (0.17, 0.20)/0.37 (0.34, 0.40)/0.25 (0.23, 0.27)	0.76 (0.75, 0.77)/0.55 (0.53, 0.57)/0.64 (0.63, 0.65)	0.51 (0.50, 0.53)
Exclude geriatrician	0.18 (0.16, 0.19)/0.34 (0.31, 0.37)/0.23 (0.21, 0.25)	0.75 (0.74, 0.76)/0.56 (0.54, 0.58)/0.64 (0.63, 0.66)	0.51 (0.50, 0.53)

### Structured data baseline

To establish a structured-data baseline, we implemented a random forest classifier trained on ICD codes, medications, and abnormal lab measurements, following prior work^12^. All features were processed using term frequency–inverse document frequency (TF-IDF) representations. The model was trained and tuned on a 90%/10% split of the full cohort after first holding out the 1,000 patients as an independent evaluation set. As shown in Table 7, our LLM-based CARE-AD framework consistently outperformed the structured-data model across all prediction horizons, achieving higher F1 scores for both AD cases and controls—particularly at earlier time points.

**Table 7:** Random forest prediction results using structured data features

Prediction year	AD cases			Controls			Accuracy
	Precision	Recall	F1 score	Precision	Recall	F1 score	
−1 day	0.45 (0.42, 0.48)	0.65 (0.62, 0.68)	0.53 (0.50, 0.56)	0.89 (0.87, 0.91)	0.78 (0.75, 0.81)	0.83 (0.81, 0.85)	0.75 (0.72, 0.78)
−1 year	0.37 (0.34, 0.40)	0.57 (0.54, 0.60)	0.45 (0.42, 0.48)	0.86 (0.84, 0.88)	0.73 (0.70, 0.76)	0.79 (0.76, 0.82)	0.70 (0.67, 0.73)
−2 year	0.31 (0.28, 0.34)	0.51 (0.48, 0.54)	0.39 (0.36, 0.42)	0.84 (0.82, 0.86)	0.69 (0.66, 0.72)	0.76 (0.73, 0.79)	0.65 (0.62, 0.68)
−3 year	0.20 (0.18, 0.22)	0.38 (0.35, 0.41)	0.26 (0.23, 0.29)	0.77 (0.74, 0.80)	0.58 (0.55, 0.61)	0.66 (0.63, 0.69)	0.53 (0.50, 0.56)
−5 year	0.18 (0.16, 0.20)	0.35 (0.32, 0.38)	0.24 (0.21, 0.27)	0.76 (0.73, 0.79)	0.55 (0.52, 0.58)	0.64 (0.61, 0.67)	0.51 (0.48, 0.54)
−7 year	0.14 (0.12, 0.16)	0.31 (0.28, 0.34)	0.20 (0.18, 0.22)	0.72 (0.69, 0.75)	0.49 (0.46, 0.52)	0.58 (0.55, 0.61)	0.45 (0.42, 0.48)
−10 year	0.11 (0.09, 0.13)	0.26 (0.23, 0.29)	0.16 (0.14, 0.18)	0.68 (0.65, 0.71)	0.44 (0.41, 0.47)	0.53 (0.50, 0.56)	0.40 (0.37, 0.43)

## Discussion

In this study, we propose CARE-AD, a novel and feasible multi-agent LLM-based framework for early AD prediction using real-world longitudinal clinical notes. Building on advancements in multi-agent systems such as MEDAGENTS^35^, our approach simulates a multidisciplinary diagnostic process where specialized agents analyze distinct aspects of AD-related signs and symptoms—cognitive impairment, physiological changes, neuropsychiatric symptoms, and other subtle indicators—extracted from clinical narratives. By dividing responsibilities across agents, the system identifies domain-specific markers that may be overlooked by a single general-purpose model. To our knowledge, this is one of the first applications of LLMs that not only extract AD-relevant indicators exclusively from unstructured clinical text but also employ a multi-agent workflow for early AD detection. Evaluations on retrospective clinical data suggest that CARE-AD offers improvements in predictive performance, helping bridge the gap between general-purpose LLM capabilities and the specialized requirements of AD-focused clinical applications.

CARE-AD outperformed single-model zero-shot approaches in our retrospective evaluation. With an accuracy of 0.53 at 10 years prior to ICD-based diagnosis, these findings suggest that relevant risk indicators may appear earlier than traditionally recognized, potentially offering a window for earlier clinical attention. While iterative single-model methods, such as self-consistency and self-refine, exceeded the zero-shot baseline, they still underperformed compared to the multi-agent strategy. The strength of CARE-AD lies in its distributed expertise and collaborative decision-making framework. Unlike self-refine and self-consistency methods, which constrain multiple reasoning paths within a single model, CARE-AD assigns distinct roles to specialized “doctor” agents, each leveraging domain-specific knowledge, and integrates their assessments through an AD specialist agent. This structure emulates real-world clinical collaboration and supports more comprehensive risk evaluation. For example, as detailed in Supplementary Note 6, the primary care physician agent identified comorbidities and the absence of cognitive screening; the neurologist agent emphasized past transient ischemic attacks and medication interactions; the geriatrician agent noted age-related vulnerabilities and polypharmacy; the psychiatrist agent highlighted how depressive symptoms could mask early cognitive decline; and the clinical psychologist agent recommended further mood and cognitive monitoring. The AD specialist agent then synthesized these insights and proposed that the patient may be experiencing cognitive decline consistent with early-stage AD, recommending confirmatory evaluations. By integrating complementary perspectives across clinical domains, CARE-AD offers an approach for evaluating early cognitive risk in a manner inspired by multidisciplinary consultation. The multi-agent design also enhances interpretability by revealing intermediate reasoning steps and showing how differing viewpoints are synthesized. While further prospective validation is needed, this approach offers a potential pathway for improving early detection, supporting longitudinal monitoring, and informing targeted interventions.

We also compared CARE-AD with an AutoGen-based multi-agent setup, which offers a general-purpose framework for inter-agent dialogue. When constrained to the same number of LLM calls (six), AutoGen underperformed relative to CARE-AD. This may be due to AutoGen’s generalized architecture, which includes predefined system messages and automated coordination mechanisms that introduce additional reasoning steps or role negotiations that are less aligned with the streamlined requirements of clinical inference. In contrast, CARE-AD’s role-specific prompting explicitly enforces task specialization, enabling more efficient extraction and synthesis of patient information. Increasing the number of LLM calls in AutoGen to 12 or 18 yielded comparable performance to CARE-AD, though improvements plateaued beyond 12 calls, indicating diminishing returns with further computation. These results suggest that CARE-AD offers a more resource-efficient alternative for early AD risk prediction.

In comparison with a traditional random forest model trained on structured EHR data, CARE-AD demonstrated the value of analyzing unstructured clinical narratives for identifying early AD risk indicators. Structured data, such as ICD codes, medications, and lab results, typically capture downstream diagnoses or late-stage manifestations, potentially missing earlier behavioral or cognitive changes. In contrast, narrative notes often contain subtle, pre-diagnostic observations that precede formal diagnosis by years. By leveraging this unstructured information, CARE-AD detected early symptom patterns more effectively than the structured-data model, particularly at longer prediction horizons. These findings reinforce the potential of LLMs in mining free-text EHR data for early disease signal detection.

This study has several important limitations. First, we relied on VHA data, which may not fully represent the broader population, as VHA patients often have distinct demographic characteristics, including a significant sex imbalance, socioeconomic challenges, and higher rates of post-traumatic stress disorder and traumatic brain injury. Consequently, our findings require validation in non-VHA populations. Second, to ensure sufficient information for prediction, we required a minimum of 5 years of longitudinal notes in the observation window. This requirement may have introduced selection bias, as patients with lower hospital utilization and fewer clinical visits—those who could benefit most from large-scale screening—were underrepresented. In future work, we plan to include additional data sources to capture this group and improve our predictive models. Third, we defined the diagnosis date using the first recorded AD-related ICD code and included a −1 day prediction window, consistent with prior studies^12^. However, manual review revealed that in some cases, the actual diagnosis may have preceded the ICD code date, potentially inflating performance estimates, particularly at the −1 day window. Including a broader range of earlier time points like −1 year, −2 years, and −3 years before diagnosis, helps better assess the model’s predictive performance across different stages of disease progression. Fourth, despite leveraging extensive baselines for comparison, privacy constraints prevented us from evaluating our approach using other cutting-edge LLMs (e.g., the GPT family^29^), limiting our ability to examine its generalizability to larger models. Nevertheless, our findings offer meaningful insights into how well the method adapts when data confidentiality is strictly enforced. In future work, we will explore publicly available datasets to more thoroughly assess how the model can scale and perform with other LLMs.

Expert-level performance in complex medical tasks like AD diagnosis will likely require collaborative, multi-agent systems. CARE-AD illustrates this approach's potential by leveraging coordinated specialized LLM agents to extract symptoms, assess risk, and predict AD onset up to 10 years before diagnosis, achieving higher accuracy than single-model baselines in our evaluation. The data extraction agent is designed to operate with longitudinal EHR data and could, with further validation, support symptom tracking and trend analysis for clinical decision-making. By incorporating domain-specific expertise, specialist agents enhance clinical decision-making, ensuring a more comprehensive and accurate assessment that may improve diagnostic interpretability. While this work focuses on AD, the underlying framework demonstrates the potential of multi-agent LLM solutions for addressing other complex medical conditions. It may be adaptable to other multifactorial diseases that require multidisciplinary expertise for diagnosis and management, providing a foundation for further exploration of AI-assisted clinical decision support.

## Methods

### Data sources and ethical approval

This study used the EHR database from the VHA Corporate Data Warehouse (CDW), covering the period from 2000 to 2022. The VHA is the largest integrated healthcare network in the U.S., comprising over 1200 medical centers and clinics, with extensive data on demographics, medications, diagnoses, procedures, clinical notes, and billing information, making it a valuable resource for large-scale health research. This study was approved by the Institutional Review Board of the US Veterans Affairs (VA) Bedford Health Care and conducted in accordance with the principles of the Declaration of Helsinki. A waiver of informed consent was obtained due to minimal risk to participants.

### Cohort design

To construct the study cohort, we adopted a case-control design prioritizing diagnostic specificity to capture biologically homogeneous AD cases suitable for identifying early predictive markers. AD cases were defined based on the presence of AD-specific ICD codes (Supplementary Table 2) between October 1, 2015 (ICD-10 implementation), and September 30, 2022. We required at least two AD diagnoses on separate occasions, with one diagnosis recorded in a specialty clinic such as neurology, geriatrics, geriatric patient aligned care team (GeriPACT), mental health, psychology, psychiatry, or geriatric psychiatry—provided by a provider specializing in neurology, vascular neurology, psychiatry, neuropsychology, or geriatric medicine. These clinic types are identified by Stop Codes (Supplementary Table 3), which the VHA uses to specify the type of outpatient care and the workload associated with a visit^43^. These stringent criteria exclude patients with non-AD dementia, ensuring our cohort captures true AD trajectories essential for studying decade-long preclinical predictors.

Observation windows for each AD case began at the later of the patient’s EHR initiation date or the study start date and ended at predetermined prediction time points prior to the first AD diagnosis (1 day, 1, 2, 3, 5, 7, and 10 years). A minimum observation period of 5 years was required, yielding 17,488 AD cases.

Controls were selected from VHA patients without any dementia diagnosis codes (Supplementary Table 4). Each AD case was matched with up to four controls based on age, sex, race/ethnicity, clinical utilization, Charlson Comorbidity Index (CCI), and Area Deprivation Index (ADI), following established methods^43^. The ADI was included to account for socioeconomic and environmental factors that shape health outcomes in AD, consistent with existing studies^44^. The final control cohort comprised 64,691 patients. Supplementary Fig. 1 details cohort inclusion and exclusion criteria.

AD diagnoses in this study reflect clinical practice, where diagnoses are based on cognitive and functional symptoms rather than biomarker confirmation. Thus, we use the terms “Alzheimer’s disease (AD)” and “AD dementia” interchangeably.

### Evaluation sampling

Because we employed LLMs for zero-shot evaluation and analyzing longitudinal notes is computationally intensive, we randomly selected 1000 AD cases and 3627 matched controls from the full cohort for evaluation. This subset approach aligns with conventional machine learning practices, where a portion of the data is reserved for testing, though no dedicated training set was needed in our zero-shot setting.

### Multi-agent framework

#### Taxonomy development and annotation

AD dementia exhibits a complex continuum of cognitive, behavioral, and functional signs that evolve over many years^2^. Accurately interpreting these signs in large volumes of longitudinal EHR notes is challenging. By focusing solely on real-world clinical observations in EHRs—rather than specialized cognitive assessments or AD-specific diagnostic tests—this work identifies subtle early indicators, such as forgetfulness, behavioral shifts, and functional difficulties, that might otherwise go unnoticed. We intend to seek those insights to uncover overlooked aspects of patient histories and enhance predictive accuracy. Building on existing literature^38^, domain experts crafted a novel, pragmatic taxonomy of five categories to capture the full spectrum of AD dementia signs and symptoms.

- Cognitive impairment: Captures the initial cognitive decline associated with AD, including subtle memory lapses, reduced problem-solving abilities, and difficulties in language comprehension, etc. These symptoms represent early indicators of neurodegeneration.
- Notice/of concern to others: Encompasses alterations in behavior and cognition that are noticeable and concerning to family members, close friends, neighbors, etc. Such changes signal deviations from the individual’s typical functioning and may include increased confusion, disorientation, or withdrawal from social activities, etc.
- Requiring assistance/Functional impairment: Indicates a progressive loss of independence in daily activities. Patients begin to require assistance with tasks of instrumental activities of daily living (iADLs) such as managing finances, taking medications properly, or handling household chores. As their functional abilities decline further, they may also require support with activities of daily living (ADLs), for example, personal hygiene and other basic self-care tasks.
- Physiological changes: Includes physical symptoms indicative of AD progression, such as hearing/smelling loss, disrupted sleep patterns (e.g., insomnia or excessive sleepiness), inability to combine muscle movements, etc.
- Neuropsychiatric symptoms: Encompasses a range of psychiatric and behavioral manifestations seen in AD. While many of these symptoms—such as mood disturbances (depression, anxiety), psychotic features (hallucinations, delusions), agitation, and aggression—tend to become more pronounced in the later stages, certain issues like depression can emerge even before an AD diagnosis is formally made.

Detailed definitions for each category are provided in the expert-curated annotation guidelines in Supplementary Note 2. Detecting these signs and symptoms from EHRs is a crucial task for early diagnosis, treatment, and care planning of AD.

To create a gold-standard dataset, we applied our proposed taxonomy by systematically annotating 5112 longitudinal EHR notes from 76 individuals with AD (excluded from the evaluation set). Under two physicians’ supervision, two trained medical professionals identified relevant sentences and assigned taxonomy-based labels. First, both annotators independently labeled all notes from six patients to assess inter-annotator reliability, achieving a high Cohen’s κ (0.868) once disagreements were resolved through discussion. They then split the remaining patients between them for annotation, consulting their supervising physicians for any ambiguous cases. This process yielded a gold-standard dataset of 11,571 sentences, demonstrating the taxonomy’s consistent applicability to clinical text.

Building on previously validated synthetic data resources shown to enhance model performance^38^, we employed a subset of an existing synthetic dataset, selecting only the symptom categories relevant to AD. The synthetic data was originally generated using two established methods: (1) a data-to-label approach, in which sentences were randomly sampled from MIMIC-III discharge summaries and annotated by a LLM guided by clinical annotation guidelines; and (2) a label-to-data approach, where GPT-4 was prompted with predefined symptom category definitions to generate synthetic clinical note sentences paired with corresponding labels. These approaches enabled the creation of diverse and high-quality training samples without manual annotation. Statistics of the dataset used in this study are provided in Supplementary Table 1.

#### LLM fine-tuning for data extraction agent

Using both annotated and synthetic datasets, we fine-tuned the LLaMA 3.1 8B Instruct model with Low-Rank Adaptation (LoRA) to develop a specialized data extraction agent^45^. This LoRA strategy substantially decreases the number of trainable parameters, thereby improving efficiency and reducing costs—key factors in large-scale, aging-focused research. At inference, LoRA’s lightweight parameter updates merge seamlessly with the base model to yield the final adapted system. We employed the Parameter-Efficient Fine-Tuning (PEFT) package^46^ to complete the fine-tuning process using 8× NVIDIA A6000 (48 GB) GPUs over approximately 10 h. Parameter settings are provided in Supplementary Table 5.

Fine-tuning was performed for logit-based classification tasks. For binary classification, the input was a single sentence, and the output was a logit-based prediction indicating whether it was AD-relevant. We used a combination of annotated and synthetic AD-relevant sentences as positive samples, and randomly sampled non-AD-relevant sentences from the longitudinal notes of the same 76 patients to form negative samples, using a 5:1 negative-to-positive ratio^38^. For multi-label classification, we used only AD-relevant sentences, and the model produced a probability distribution over predefined AD symptom categories, with the predicted category selected via an argmax over logits.

We developed the CARE-AD framework using the LLaMA 3.1 8B and 70B models. The data extraction agent was fine-tuned using the LLaMA 3.1 8B model to balance performance and computational efficiency, enabling training on clinical data with manageable resource demands. The specialty doctor agents and the AD specialist agent were implemented using the LLaMA 3 70B model, selected for its strong zero-shot and in-context reasoning capabilities, scalability, and open-source availability—allowing secure deployment within the VINCI environment in compliance with VA data governance policies. Proprietary models such as GPT-4 were excluded due to VHA privacy restrictions prohibiting data transfer outside the VINCI system. While medical-domain LLMs (e.g., BioGPT^47^, MedAlpaca^48^, PMC-LLaMA^49^, Clinical Camel^50^) may offer domain-specific advantages, they were not adopted due to limitations in scale, training data (mostly biomedical literature rather than real-world EHR notes), or deployment restrictions.

#### Patient time-series construction

To generate patient profiles suitable for temporal modeling of AD progression, we aligned each patient’s clinical notes to their age at the time of each visit. We applied the fine-tuned LLaMA 3.1 8B model to classify sentences into one of the predefined AD symptom categories. The categorized sentences were then aggregated chronologically to create structured, time-stamped profiles capturing symptom evolution over time. These profiles enabled the specialist agents to assess patients’ longitudinal trajectories rather than isolated encounters, facilitating temporally informed risk assessments.

#### Domain-specific and AD specialist agents

To emulate expert clinical reasoning without additional fine-tuning, we implemented structured, role-specific prompts within a multi-agent framework. Five domain-specific agents—a primary care physician, neurologist, geriatrician, psychiatrist, and clinical psychologist—were each guided by prompts reflecting their respective clinical expertise. These agents evaluated patient symptom profiles and provided domain-specific assessments. An AD specialist agent then integrated these evaluations with the extracted evidence to estimate the likelihood of AD development. Supplementary Table 6 outlines the agent configurations within the CARE-AD framework, and the full set of prompts is provided in Supplementary Note 5.

#### Baseline comparisons

To establish a conversational multi-agent baseline, we implemented the AutoGen framework using the LLaMA 3 70B model. The system comprised a supervisor agent (AD specialist) and five domain-specific agents—primary care physician, neurologist, psychiatrist, geriatrician, and clinical psychologist—each guided by structured prompts reflecting their clinical expertise. Agents engaged in multi-round dialogues to assess shared patient profiles, critique each other’s reasoning, and iteratively refine their outputs under the supervision of the AD specialist. We evaluated the system’s performance across varying numbers of dialogue rounds. The prompts used for the LLM-based baselines are provided in Supplementary Note 7, and Supplementary Table 7 summarizes all baseline model configurations and comparisons.

For the structured-data baseline, we implemented a random forest classifier using scikit-learn^51,52^. This model was chosen based on prior evidence that random forests outperform logistic regression for structured-data-based AD prediction^12^. We used structured EHR features--ICD diagnosis codes, medications, and abnormal lab measurements--processed with term frequency–inverse document frequency (TF-IDF) representations to enhance discriminative power. Additional implementation details are provided in Supplementary Note 8.

#### Data preprocessing

During the study period (2000–2022), we examined unstructured EHR notes from each patient’s EHR initiation date or the study start date, whichever was later, up to their first ICD-coded AD diagnosis (the AD index date). To manage computational demands across this 20-year span, we first restricted analysis to notes from clinically relevant encounter types, including primary care, emergency visits, home-based primary care (HBPC), memory clinics, neurology, neuropsychology, geriatrics, psychiatry, psychology, cognitive care nursing, mental health clinics, compensation and pension examinations, and consultation visits.

To prepare unstructured text for sentence-level classification, we applied standard pre-processing steps. Sentence segmentation was performed using spaCy^53^, which parsed clinical narratives into individual sentences. We then applied basic sentence filtering heuristics to remove low-information or noisy inputs, such as those with fewer than three tokens, numeric-only content, or more than 125 tokens. These pre-processing steps ensured cleaner inputs and more consistent inference performance when using LLMs.

#### Evaluation and performance metrics

For evaluation, in cases where the AD specialist agent did not provide a definitive “Yes” or “No” response—typically recommending further clinical evaluation instead—we applied a consistent evaluation rule. Specifically, if the agent explicitly stated there was no AD risk or that symptoms were not related to AD, the case was classified as non-AD. All other responses, including expressions of uncertainty or deferrals for further testing, were classified as AD-positive, aligning with the study’s goal of identifying early, pre-diagnostic risk indicators. This protocol reflects the clinical reality that early signs of AD often emerge before formal diagnostic confirmation.

To quantify model performance, we used stratified bootstrapping with 5000 iterations to estimate 95% confidence intervals (CIs) for CARE-AD and all baseline models. In each iteration, we resampled the test set with replacement while preserving the original AD/control class distribution and computed performance metrics. The 95% CI was calculated by taking the 2.5th and 97.5th percentiles of the resulting metric distribution.

## Supplementary information

Supplementary Information

## Data Availability

The data used in the preparation of this article are from VHA. Approval by the Department of Veterans Affairs is required for data access.

## Code availability

Code for prediction models can be made available upon request. Relevant packages include PEFT, LLaMa 3.1 8B instruct^53,54^, LLaMa 3 70B^55^, Autogen, and Scikit-learn^52^. All prompts used in this work are in the supplementary.
