# Supplementary material for: CARE-AD: a multi-agent large language model framework for Alzheimer’s disease prediction using longitudinal clinical notes
Source: NPJ Digit Med. 2025 Aug 24;8:541. doi: 10.1038/s41746-025-01940-4 (PMC12375024; doi:10.1038/s41746-025-01940-4)
Supplement: Supplementary file 1 — Supplementary Information [file 41746_2025_1940_MOESM1_ESM.pdf]

Supplementary

CARE-AD: A Multi-Agent Large Language Model Framework for Alzheimer's Disease Prediction Using Longitudinal Clinical Notes

Supplementary Figure 1. Cohort generation flowchart.

Supplementary Table 1. Dataset statistics used for fine-tuning data-extraction agent.

Supplementary Table 2. AD-related ICD code description.

Supplementary Table 3. AD-related stop codes within VHA.

Supplementary Table 4: ICD codes used to exclude patients with dementia when selecting controls.

Supplementary Table 5. Parameters for data extraction agent: fine-tuning LLaMa 3.1 8B Instruct on 8x A6000 48 GB GPUs.

Supplementary Table 6. Configuration of agents in the CARE-AD Framework.

Supplementary Table 7. Model Comparison: Architectures, Key Techniques, Performance, Strengths, and Limitations

Supplementary Note 1. CARE-AD system overview and technical architecture.

Supplementary Note 2. Annotation guidelines for identifying AD dementia signs and symptoms.

Supplementary Note 3. Data time series construction process.

Supplementary Note 4. Illustrative example of patient-level profile aggregation

Supplementary Note 5. Setup and prompts for the agents in CARE-AD.

Supplementary Note 6: Inputs excerpts and outputs of Doctor Agents for an example patient in the CARE-AD Framework.

Supplementary Note 7. Prompts for baseline models.

Supplementary Note 8. Random forest model implementation details.

Supplementary Figure 1. Cohort generation flowchart.

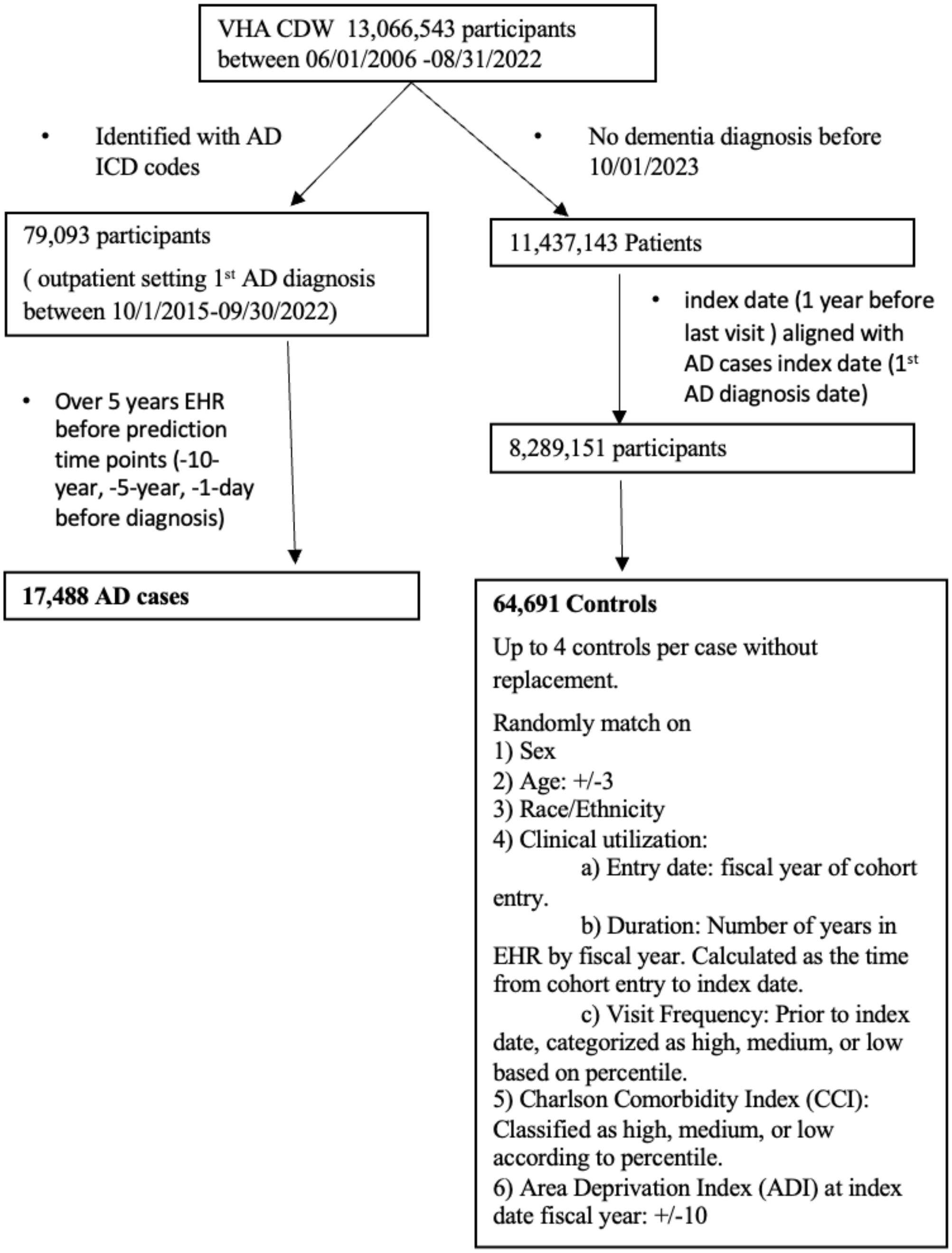

Supplementary Table 1. Dataset statistics used for fine-tuning data-extraction agent.

Category	Gold	Synthetic
Cognitive impairment	6240	4082
Notice/concern by others	785	2076
Require assistance	1864	1819
Physiological changes	1340	7487
Neuropsychiatric symptoms	1342	1626

Supplementary Table 2. AD-related ICD code description.

ICD Code	Description
331·0 (ICD-9)	Alzheimer’s disease
G30.0 (ICD-10)	Alzheimer’s disease with early onset
G30.1 (ICD-10)	Alzheimer’s disease with late onset
G30.8 (ICD-10)	Other Alzheimer’s disease
G30.9 (ICD-10)	Alzheimer’s disease, unspecified

Supplementary Table 3. AD-related stop codes within VHA.

Stop Code	Description
320	Dementia clinic (discontinued in Oct 2021)
315	Neurology
318	Geriatric problem-focused consultation clinic (including memory clinic)
350	Geriatric primary care
538	Psychological testing
502	Mental health clinic
576	Psycho-geriatric clinic
509	Psychiatry

Supplementary Table 4: ICD codes used to exclude patients with dementia when selecting controls.

Diagnostic Category	ICD codes
Alzheimer's Disease	331.0, G30.0, G30.1, G30.8, G30.9
Non-Specific Dementia/ Related Dementia / Other Dementias	294.20, F03.90: Unspecified dementia without behavioral disturbance<br>294.21, F03.91: Unspecified dementia with behavioral disturbance<br>290.0: Senile dementia uncomplicated<br>290.20: Senile dementia with delusional features<br>290.21: Senile dementia with depressive features<br>290.3: Senile dementia with delirium<br>331.2, G31.1 Senile degeneration of the brain<br>331.6: Corticobasal degeneration<br>331.7: Cerebral degeneration in diseases classified elsewhere<br>331.9: Cerebral degeneration, unspecified<br>331.89: Other cerebral degeneration<br>330.9: Unspecified cerebral degeneration in childhood<br>797: Senility without mention of psychosis<br>G31.9: Degenerative disease of nervous system, unspecified<br>290.40, 290.41, 290.42, 290.43, F01.50, F01.51, F01.511, F01.518, F01.52, F01.53, F01.54: Vascular dementia<br>331.1, 331.19, G31.0, G31.09: Frontotemporal/Other Fronto-Dementia<br>331.82, G31.83: Lewy body dementia<br>290.10, 290.10, 290.11, 290.12, 290.13, G30.0: Presenile/Early onset dementia<br>331.5, G91.2: Idiopathic Normal Pressure Hydrocephalus<br>046.1, A81.00, A81.01, A81.09: Creutzfeldt-jakob disease<br>331.11, G31.01: Pick's disease<br>F10.96 Korsakoff Syndrome<br>294.10, 294.11: Dementia in conditions classified elsewhere with/without behavioral disturbance.<br>F02.80, F02.81, F02.811, F02.818, F02.82, F02.83, F02.84: Dementia in other diseases classified elsewhere with/without behavioral disturbance<br>046.3, A81.2: Progressive multifocal leukoencephalopathy<br>046.79: Other and unspecified prion disease of central nervous system<br>291.1: Alcohol-induced persisting amnestic disorder

	291.2: Alcohol-induced persisting dementia<br>292.82: Drug-induced persistent dementia<br>292.83: Drug-induced persistent amnestic disorder<br>294.0: Amnestic disorder in conditions classified elsewhere<br>799.55: Frontal lobe and executive function deficit<br>G31.2: Degeneration of nervous system due to alcohol<br>G31.85: Corticobasal degeneration<br>G31.89: Other specified degenerative diseases of nervous system<br>G13.2, G13.8: Systemic atrophy primarily affecting the central nervous system in myxedema<br>G94: Other disorders of brain in diseases classified elsewhere<br>R41.840, R41.841, R41.842, R41.843, R41.844: Other specified cognitive deficit<br>R54: Age-related physical debility<br>F04: Amnestic disorder due to known physiological condition<br>F05: Delirium due to known physiological condition<br>F10.7, F11.7, F12.7, F13.7, F14.7, F15.7, F16.7, F18.7, F19.7, F10.6, F11.6, F12.6, F13.6, F14.6, F15.6, F16.6, F18.6, F19.6: Mental and behavioural disorders<br>A81.89: Other atypical virus infections of central nervous system<br>I67.3: Progressive vascular leukoencephalopathy<br>R41.81: Age-related cognitive decline
Mild Cognitive Impairment (MCI)	331.83, G31.84

Supplementary Table 5. Parameters for data extraction agent: fine-tuning LLaMa 3.1 8B Instruct on 8x A6000 48 GB GPUs.

LoRA parameters	
LoRA-alpha	16
LoRA-dropout	0.05
LoRA-rank	8
bias	None
Other parameters	
num_train_epochs	1
per_device_train_batch_size	8
Gradient_accumulation_steps	4
optimizer	Paged_adamw
Learning_rate	1e-4
Tf32	True
Max_grad_norm	0.3
Warmup_ratio	0.01
Max_length	4k
Lr_scheduler_type	cosine

Supplementary Table 6. Configuration of agents in the CARE-AD framework.

Agent Name	Model	Input	Output
Data Extraction Agent	Llama 3.1 8B<br>(Fine-tuned on AD-related signs and symptoms data)	Individual sentences from clinical notes.	Probability scores for each symptom category
Primary care physician agent	LlaMA 3 70B with zero-shot prompts	Patient AD Profile, constructed by aligning AD-related symptom mentions to patient age and categorizing them by symptom type.	Assessment from primary care perspective
Neurologist agent			Neurological assessment
Geriatrician agent			Geriatric assessment
Psychiatrist agent			Psychiatric assessment
Clinical Psychologist agent			Assessment from clinical psychologist
AD Specialist	LlaMA 3 70B with zero-shot prompts	Patient AD profile + all analyses from specialist agents	Final AD diagnosis ("Yes"/"No") with rationale

Supplementary Table 7. Model Comparison: Architectures, Key Techniques, Performance, Strengths, and Limitations

Method	System Architecture	Key Techniques	Performance	Strength	Limitations
Care-AD (Proposed)	Multi-agent system for diagnostics	Specialized agent roles, Task-specific communication, Domain-optimized reasoning chains	Best overall	Domain-specific optimization Robust multi-perspective analysis High diagnostic accuracy Interpretable reasoning process	Higher computational cost, Task-specific design limits generalizability
AutoGen (similar agent design)	General-purpose multi-agent framework	User-defined agents, Flexible memory, Configurable communication	Good when optimized	High flexibility, Customizable Scalable architecture	Requires extensive configuration, Not optimized for specific domains, Performance depends on agent design
Zero-shot	Single agent	Clear and direct instruction	Baseline	Simple and efficient, No additional infrastructure, Fast inference	Poor handling of complex cases, No error correction mechanism, Incapable with multi-faceted problems
Chain of Thought (CoT)	Single agent with explicit reasoning	Chain-of-thought prompting, Step-by-step problem decomposition	Moderate improvement	Straightforward and step-by-step explainable	Limited handling of complex cases, No error correction mechanism, Struggles with multi-faceted problems
Self-refine	Iterative single-agent system	Multi-round self-review, Error detection and correction, Iterative refinement process	Moderate improvement	Self-correction, Accuracy gains over zero-shot/CoT systems, error reduction	Limited perspective and diversity, Diminishing returns after iterations

Self-consistency	Ensemble of single-agent runs	Multiple reasoning paths, Majority voting	Moderate improvement	Increased reliability, Better uncertainty handling, Error reduction via consensus	Still single perspective, No collaborative problem-solving

Supplementary Note 1. CARE-AD system overview and technical architecture.

The system comprises three main components:

1. Data Extraction Agent
2. Specialist Agents (5 in total)
3. AD Specialist Agent

For each patient with longitudinal EHR notes, the workflow proceeds in three steps:

Step 1 – Data Extraction (Execution and Memory)

- Execution: We fine-tune a LLaMA 3.1 8B model to perform sentence-level classification, identifying sentences that mention AD-related signs and symptoms.
- Memory: The outputs of this step (sentence-level labels) are stored temporarily to build an age-aligned symptom profile for the patient.
- Reasoning: The data extractor uses its trained model weights to classify sentences but does not employ explicit chain-of-thought prompting.
- Output: A structured profile of extracted AD-related observations aligned by patient age.

Step 2 – Specialist Agents (Reasoning and Execution)

- Execution: Five specialist agents (implemented using LLaMA 3 70B) independently analyze the age-aligned symptom profile. Each specialist focuses on a predefined domain (e.g., neurology, geriatrics, psychiatry).
- Reasoning: The analysis is guided by structured prompts that instruct each agent to assess AD risk from their domain perspective. This internal reasoning does not use external chain-of-thought or multi-step plans.
- Memory: Each specialist receives the same patient profile as input. No persistent memory exists across different patients.
- Output: Five domain-specific assessments summarizing AD-related concerns.

Step 3 – AD Specialist Agent (Integration and Reasoning)

- Execution: The AD specialist agent (LLaMA 3 70B) synthesizes the original patient profile and the five specialist assessments to produce a final AD risk prediction.
- Reasoning: Structured prompts guide this agent to weigh domain insights and provide an integrated conclusion.
- Memory: As in prior steps, outputs are passed sequentially, and no long-term memory is retained.
- Output: The final AD risk decision.

Planning

- Unlike dynamic planning-execution systems that rely on a planner agent to generate case-specific plans, our approach uses a predefined, deterministic workflow tailored for AD risk assessment under resource constraints. This design simplifies reproducibility. As an extension, we plan to incorporate a planner agent in future work to enable personalized assessment strategies.

Chain of Thought

- In this study, we do not explicitly implement chain-of-thought (CoT) reasoning within the agents. Each agent performs single-step reasoning guided by structured prompts.

Memory

- Memory is implemented as short-term transfer between steps: outputs from the data extraction agent are passed to the specialist agents and then to the AD specialist agent. No information is preserved across patient cases.

Execution

- Execution is operationalized via sequential LLM calls:
  - Step 1: Each sentence is classified individually.
  - Step 2: The constructed profile is analyzed in parallel by specialist agents.
  - Step 3: The AD specialist integrates prior outputs to produce the final risk assessment.

Supplementary Note 2. Annotation guidelines for identifying AD dementia signs and symptoms

Class 1: **Cognitive impairment**

Cognitive impairment is when a person has trouble remembering, learning, concentrating, or making decisions that affect their everyday life, including patient subjective statements as well as Dr. statements.

- Forgetting appointments and dates.
- Forgetting recent conversations and events.
- Having a hard time understanding directions or instructions.
- Losing your sense of direction.
- Losing the ability to organize tasks.
- Becoming more impulsive.
- Memory loss.
- Frequently asking the same question or repeating the same story over and over (perseveration)
- Not recognizing familiar people and places
- Having trouble exercising judgment, such as knowing what to do in an emergency
- Difficulty planning and carrying out tasks, such as following a recipe or keeping track of monthly bills
- meaningless repetition of own words, lack of restraint, wandering and getting lost
- lose your train of thought or the thread of conversations
- trouble finding your way around familiar environments
- problems with speech or language
- mental decline, difficulty thinking and understanding, confusion in the evening hours, disorientation, lack of orientation, forgetfulness, making things up, mental confusion, difficulty concentrating, inability to create new memories, inability to do simple math, or inability to recognize common things, poor judgment, impaired communication, poor concentration, difficulty remembering recent conversations, names or events
- forget things more often, forget important events such as appointments or social engagements, issues with recall, changes in abstract reasoning ability

Class 2: **Notice/concern by others** (not by provider, refers to friends/family and neighbors)

family complains of X (may be related to any class including cognitive decline, functional impairment, Neuropsychiatric and physiological changes)

- noticed changes in ability, speed
- concern expressed by family/friends
- complaints of patient easily angered
- Daughter reports that she repeatedly asks the same question...had difficulties using her smartphone.
- Daughter reports that she has issues with banking...some decrease in personal hygiene, forgets to take meds, forgets where food is in the house, etc.

- Patient has gone out at 1:30 a.m. without telling anyone; they are concerned, but patient always has a response.
- She (daughter) tells me that her mom has repeatedly changed the medications in the pill boxes that she has arranged for her.

Class 3: **Requires assistance** (defined as from a person)/ functional impairment

- needs help with or loss of ability with ADLs/iADLs, difficulty with self-care, trouble managing belongings
- ADLs: dressing, eating, toileting, bathing, grooming, mobility
- iADLs: housekeeping-related activities (cleaning, cooking, and laundry) and complex activities (telephone use, medication intake, use of transportation/driving, budget/finance management, and shopping)
- The patient will continue to require assistance with all complex medical, legal and financial decision making.
- She will need 24-hour supervision for her safety.
- Direct supervision is required for medications using a pillbox.
- Best not to have him use stove.
- If left alone for period of time, will need guardian alert or consider camera surveillance.
- He is able to make a meal, to dress himself, to bathe, to shave, but continues to need help with finances.
- Wife has to remind him about appointments, in particular.
- Driving should not be permitted, and he will need assistance with IADLs and decision making.
- Veteran does need assistance with all IADLs and most ADLs.
- Traveling out of neighborhood, driving, arranging to take buses-limited night driving now
- Resides in assisted living facility or nursing home
- Writing checks, paying bills, balancing checkbook-minimal (automatic payment) N/A
- Playing a game of skill-no hobbies N/A

Class 4: **Physiological changes**

- senses: vision, hearing, smell loss, SNHL: sensorineural hearing loss, HoH,
- sleep: Excessive daytime sleepiness, changes in sleep patterns
- speech/swallowing (speech difficulties also in "Cognitive Impairment" class)
- movement/gait/balance
- inability to combine muscle movements: jumbled speech, difficulty speaking, aphasia, dysphasia, difficulty swallowing, dysphagia, difficulty walking, mobility, problems with gait and balance, gait slowing

(Focus on early possible associations, late physiological changes associated with AD like seizure, incontinence etc. are skipped.)

Class 5: **Neuropsychiatric symptoms**

- mood changes: depression, irritability, aggression, anxiety, apathy, personality changes, behavioral changes, agitation, short-temper, quick to anger, anger issues, mood instability, hypervigilance, negative cognitions, mood lability, labile mood, anhedonia
- feeling increasingly overwhelmed by making decisions and plans
- paranoia, delusions, hallucinations, psychosis, hear voices, see X

Supplementary Note 3. Data time series construction process.

1. Note selection and temporal alignment

For each patient, we first extract all clinical notes of selected types within the observation period. Each note is aligned to the patient's age at the date of the visit, resulting in a chronological mapping of the clinical documentation. For example:

Patient ID: 1000xxxxxx

Age	Note
…	…
64	Note_ID: 1000172xxxxxx, 1000175xxxxxx, …
65	Note_ID: 1009356xxxxxx, …
66	Note_ID: 1032800xxxxxx, …
…	…

2. Sentence-Level Annotation

We then use our fine-tuned LLaMA 3.1 8B model to classify each sentence in every note by AD-related symptom category. This step yields structured annotations of symptoms, organized by patient age.

3. Age-Aligned Profile Assembly

The outputs are aggregated to form an age-aligned symptom profile, which reflects how signs and symptoms evolve over time. An excerpt of a de-identified example is shown below:

Patient ID: 1000xxxxxx

Age	AD-related signs/symptoms
…	…
64	Cognitive impairment:
	Notice/concern by others:
	Physiological changes:<br>Knees are gone, the plastic is worn out.<br>Ambulates with cane<br>Lower lip laceration X xx hrs.
	…
65	Cognitive impairment:<br>… seems pretty confused about what to eat …<br>… continue utilizing the cognitive behavioral therapy techs and discussed with …
	Notice/concern by others:<br>… he had troubles coping with it and had suicidal ideas.

	Per his wife, … have difficulty recalling appts …
	Physiological changes:<br><br>… the results were primarily that of his H/H with reflux with recommendations made for …<br><br>It seems his mood has been under the control with the xxx mg sertraline.<br><br>A few fibrous changes radiating form the hilar regions.<br><br>…
	…
66	…
…	…

4. Input to Specialist Agents

This structured, temporally ordered profile is then provided as input to each specialist agent in the next stage of the workflow. Each agent receives a prompt instructing it to analyze the patient's longitudinal symptom progression across ages, rather than isolated time points, supporting temporally contextualized reasoning.

Supplementary Note 4. Illustrative example of patient-level profile aggregation

A patient profile consists of two components: (1) demographic information, including sex, birth year, race, and ethnicity; and (2) a list of age-specific sentences describing AD-related signs and symptoms extracted from clinical notes.

Patient Profile for Pt ID xxxxxxx:

Demographic Information

- **Sex**: Female
- **Birth Year**: 19xx
- **Race**: Asian
- **Ethnicity**: Not Hispanic

AD-related signs and symptoms by Age

Age 67:

- [Cognitive Impairment]: Pt reports occasionally forgetting grocery lists; spouse notes she asked about weekend plans twice in one day.
- [Physiological Changes]: Mild hearing loss in left ear per audiology consult; denies impact on daily life.
- [Notice by Others]: Son states pt "misplaced passport before trip" and "anxious about airport directions".
- [Cognitive Impairment]: Pt describes trouble recalling new coworker names: "They tell me, but it slips away".

Age 68:

- [Neuropsychiatric Symptoms]: increased irritability when routines are disrupted: "snaps if dinner is late".
- [Cognitive Impairment]: Pt mentions difficulty focusing during budget meetings: "Numbers get jumbled in my head". ;

Age 70:

- [Notice by Others]: Daughter notes pt "repeatedly checked stove knobs" before leaving home last month.
- [Physiological Changes]: Complains of muffled taste for coffee"
- [Neuropsychiatric Symptoms]: "Spouse describes apathy toward book club: 'Used to love it, now says it's too much work'."
- [Cognitive Impairment]: "Pt states: 'I keep losing track of time—missed two haircuts this year'."

...

Supplementary Note 5. Setup and prompts for the Agents in CARE-AD.

Data Extraction Agent: No prompts are used; the fine-tuned model directly outputs category probabilities, and the category with the highest probability is selected as the predicted label for the input sentence.

F**ive Specialty doctor Agent**:

These agents assess patient's situations and output concerns related to AD if there's any.

Their prompt structures are defined as follows:

You are {the agent name and role}. {Instruct the agent to assess the patient's situation and identify concerns relating to AD from the agent's domain.}

Additional instructions: {specialist-specific guidance}.

Patient information: {patient information}

Specific prompts are as follows:

1) PRIMARY CARE DOCTOR

Prompt:

You are a primary care physician with comprehensive knowledge of adult health and preventive care. A new patient has arrived for evaluation. Please assess the patient's overall physical health, medical history, comorbid conditions, and daily functioning. Identify any concerns that may be related to Alzheimer's disease, and briefly explain why these findings are concerning.

Additional Instructions:

• Highlight any red flags that warrant further neurological or cognitive evaluation.

• Summarize key clinical observations that could guide next steps in the diagnostic process.

Patient information: {patient information}

---

2) NEUROLOGIST

Prompt:

You are a neurologist specializing in disorders of the brain and nervous system. A new patient has arrived for evaluation. Please evaluate the patient's neurological status, including memory, executive function, and any motor or sensory findings. Identify concerns that may be related to Alzheimer's disease and explain the reasoning behind each concern.

Additional Instructions:

• Focus on symptoms such as memory loss, language difficulties, or other cognitive deficits.

• Provide a concise rationale for suspected AD-related signs.

Patient information: {patient information}

---

3) GERIATRICIAN

Prompt:

You are a geriatrician with expertise in the care of older adults. A new patient has arrived for evaluation. Please assess the patient's overall functional status, comorbidities, and geriatric syndromes (e.g., fall risk, frailty, polypharmacy). Identify any concerns that may be related to Alzheimer's disease and provide a brief explanation for each.

Additional Instructions:

• Consider the patient's aging trajectory, functional independence, and support system.

• Highlight interactions between comorbidities and cognitive decline.

• Integrate information about daily living activities and any noticeable changes in routine or self-care.

Patient information: {patient information}

---

4) PSYCHIATRIST

Prompt:

You are a psychiatrist specializing in mental and emotional health. A new patient has arrived for evaluation. Please evaluate the patient's psychiatric presentation, including mood, affect, and behavioral changes. Identify any concerns potentially linked to Alzheimer's disease and articulate the basis for each concern.

Additional Instructions:

• Assess depression, anxiety, or other psychiatric comorbidities that may mask or exacerbate cognitive decline.

• Note any behavioral disturbances, personality changes, or psychotic features that could be indicative of AD.

• Consider how cognitive deficits might interact with mental health conditions in formulating your overall clinical impression.

Patient information: {patient information}

---

5) CLINICAL PSYCHOLOGIST

Prompt:

You are a clinical psychologist focusing on cognitive assessment. A new patient has arrived for evaluation. Please evaluate the patient's cognitive domains (memory, attention, language, executive function, and visuospatial skills). Identify concerns that may indicate Alzheimer's disease and explain the underlying reasons for these concerns.

Additional Instructions:

• Highlight patterns of cognitive performance characteristic of Alzheimer's disease.

• Include relevant psychological theories or models that support your assessment.

Patient information: {patient information}

AD Specialist Agent: You are a highly qualified specialist in Alzheimer's disease, with extensive clinical and research experience. You are presented with a comprehensive profile of a patient's AD-related signs and symptoms, alongside evaluations from multiple specialty clinicians. Please integrate this information in light of recognized diagnostic criteria and determine whether the patient is likely to develop Alzheimer's disease in the future. Provide your assessment as either "Yes" or "No", followed by one or two concise sentences explaining the basis for your conclusion.

Patient information: {patient information}

Specialist analysis: {specialist}: {analysis}

The outputs of all agents are plain text. The output of the AD Specialist Agent is parsed by the approach mentioned in the Method section **"**in cases where the AD specialist agent did not provide a definitive 'Yes' or 'No' response—typically recommending further clinical evaluation instead—we applied a consistent evaluation rule. Specifically, if the agent explicitly stated there was no AD risk or that symptoms were not related to AD, the case was classified as non-AD. All other responses, including expressions of uncertainty or deferrals for further testing, were classified as AD-positive, aligning with the study's goal of identifying early, pre-diagnostic risk indicators**"**

Supplementary Note 6. Inputs excerpts and Outputs of Doctor Agents for an Example Patient in the CARE-AD Framework

Example prompts for doctor agents:

(Input provided to the primary care physician; personally identifiable information (PII) has been removed, and some content is replaced with ellipses (…) to protect privacy and conserve space.)

```
You are a primary care physician with comprehensive knowledge of adult health and preventive care. A new patient has arrived for evaluatio
n. Please assess the patient’s overall physical health, medical history, comorbid conditions, and daily functioning. Identify any concerns
that may be related to Alzheimer’s disease, and briefly explain why these findings are concerning.
 Additional Instructions:
• Highlight any red flags that warrant further neurological or cognitive evaluation.
• Summarize key clinical observations that could guide next steps in the diagnostic process.

Patient information:
•       Sex: Female
•       Birth Year: xxxx
•       Race: Asian
•       Ethnicity: Not Hispanic

AD-related signs and symptoms by Age

Age xx:
[Physiological Changes]: ALLERGIES: FLOWERS, PERFUME, KEFLEX
[Physiological Changes]: ACTIVE PROBLEMS:
Computerized Problem List is the source for the following:
1. Dysphagia * 05/02/00
2. Chronic bronchitis * 04/26/00 By History Ongoing For About 50 Years
...

Age xx:
[Cognitive impairment]: ... continue utilizing the cognitive behavioral therapy ...
...

Age xx:
[Notice/concern by others]:  ... need help with shopping ...
```

The inputs for other doctor agents follow a similar format, each using agent-specific prompts combined with the patient’s AD profiles. The complete list of agent-specific prompts is provided in Supplementary Note 5.

Example corresponding output from doctor agents:

From primary care physician agent

```
**Some specific concerns related to Alzheimer's disease (AD)**:
1.      Multiple comorbidities: The patient has an extensive list of active problems, including card
iovascular disease, cerebrovascular accident, depressive disorder, and others. These comorbidities c
ould potentially contribute to cognitive decline or exacerbate existing cognitive impairment.
2.      History of TIAs: The patient experienced a transient ischemic attack (TIA) in 1997, which in
creases the risk of developing dementia, including AD.
3.      Depressive disorders: The patient has a history of major depressive disorder, recurrent epis
ode, which is a known risk factor for AD. Depression can also worsen cognitive function and accelera
te cognitive decline.
4.      Polypharmacy: The patient takes multiple medications, which can lead to adverse drug interac
tions, increased fall risk, and decreased cognition.
5.      Limited mobility: The patient uses scooters, leg braces, and crutches, indicating limited mo
bility, which can impact daily functioning and independence.
6.      No obvious mention of cognitive assessment or evaluation: Despite the patient's age and como
rbidities, there is no explicit mention of cognitive assessments, such as MMSE or MoCA, being perfor
med. This raises concern about potential undiagnosed cognitive impairments.
```

From neurologist agent

```
**Concerns related to AD**
1.      History of cerebrovascular accident: TIAs can increase the risk of developing dementia, incl
uding AD.
2.      Multiple chronic conditions: The patient’s numerous comorbidities, such as coronary artery d
isease, hypertension, and chronic bronchitis, could contribute to increased cardiovascular risk, pot
entially exacerbating AD progression.
3.      Depressive episodes: Repeated experiences of major depressive disorder may indicate vulnerab
ility to cognitive decline or even accelerate AD development.
4.      Medication profile: Polypharmacy, particularly with medications like gabapentin and antidepr
essants, may influence cognitive function or interact with potential AD treatments.
While these concerns don't necessarily confirm AD diagnosis, they warrant close monitoring and consi
deration of cognitive assessments to evaluate the patient's risk profile.
```

From geriatrician agent

```
**Concerns related to AD**
1.      History of CVA: The patient experienced a transient ischemic attack (TIA) in 1997, which inc
reases the likelihood of subsequent cognitive decline or vascular dementia.
2.      Multiple Chronic Conditions: The patient's numerous comorbidities could contribute to cognit
ive slowing or decreased reserve capacity, making them potentially more susceptible to AD-related co
gnitive changes.
3.      Medication Profile: Polypharmacy, including medications like gabapentin and antidepressants,
might impact cognition, particularly if dosages need adjustment or interactions occur.
4.      Subjective Complaints: Although the patient currently denies depressive thoughts or suicidal
ideations, previous experiences suggest vulnerability to mental health issues, which could influence
cognitive performance over time.
```

From psychiatrist agent

```
**Concerns related to AD**
1.      Age: The patient falls within an age range where AD risk increases significantly.
2.      Medical Comorbidities: Presence of chronic conditions like coronary artery disease, cerebrov
ascular accident (history of transient ischemic attack), and hypertension might contribute to increa
sed risk of vascular dementia or mixed dementia (Alzheimer's+vascular).
3.      History of Depression: Repeated episodes of major depressive disorder could potentially mask
early cognitive decline associated with AD.
4.      Medication Use: Long-term use of medications such as antidepressant (sertraline, trazodone)
and gabapentin might influence cognitive performance or interact with potential AD-related changes.
5.      Limited Information: Although the patient's history suggests intact cognitive function, limi
ted information is available regarding specific cognitive assessments, memory evaluations, or behavi
oral observations that would help rule out AD.
```

From neuropsychologist agent

```
**Concerns related to AD**
1.      Cognitive Functioning: While the patient demonstrates intact cognition, insight, and judgmen
t, it is crucial to continue monitoring their cognitive abilities due to their age and medical comor
bidities.
2.      Emotional Status: The patient's reported euthymic mood and lack of suicidal/homicidal ideati
on suggest relatively stable emotions. Nevertheless, regular assessments should focus on detecting a
ny subtle changes in mood or affective states.
3.      Medical Comorbidities: Managing the patient's multiple chronic conditions effectively is vit
al to preventing potential cognitive decline or exacerbating existing conditions.
```

Output from the AD specialist agent:

Yes.
Based on the patient's history and current presentation, I believe the patient may be experiencing cognitive decline consistent with Alzheimer's disease (AD).
Several factors support this diagnosis:
1. History of cerebrovascular accident: The patient experienced a transient ischemic attack (TIA} in 1997, which increases the risk of developing dementia, including AD.
2. Multiple comorbidities: The patient has numerous chronic conditions, such as coronary artery disease, hypertension, benign prostatic hypertrophy, and others, which can contribute to cognitive impairment.
3. Reflux symptoms and dysphagia: Although managed with dietary changes and medications, these issues could indicate early signs of neurological dysfunction, potentially related to AD.
4. Psychiatric history: The patient has a history of major depressive disorder, recurrent episode, which is common among individuals with AD.
5. Current mental status evaluation: While the MSE indicates intact cognition, insight, and judgment, subtle cognitive deficits might not be immediately apparent.
Considering these points, I recommend further evaluation, including neuropsychological testing and imaging studies (e.g., MRI), to confirm or rule out a diagnosis of Alzheimer's disease.

Supplementary Note 7. Baseline Model Settings

1. zero-shot approach relying on a single LLM call

The following prompt is sent to LLM in one call to get the result.

Prompt: The following is a summary of a patient's signs and symptoms extracted from their longitudinal electronic health records. Based on this information, please assess whether the patient is likely to develop Alzheimer's disease (AD). Provide a binary prediction ("Yes" or "No") followed by a brief justification for your decision.

Patient information: {patient information}

The output is plain text and is parsed using the same approach as in the proposed method.

2. A self-consistency approach that improves reasoning accuracy by generating multiple diverse response and selecting the most consistent output through majority voting or confidence-based aggregation,

In a self-consistency system, the LLM generates multiple reasoning paths, and the final output is selected based on a review of all generated paths.

It operates according to the following mechanism:

Reasoning Prompt: The following is a summary of a patient's signs and symptoms extracted from their longitudinal electronic health records. Based on this information, please reason through the likelihood of the patient developing Alzheimer's disease (AD).

Patient information: {*patient information*}

The output is plain text which will be fed to the summarization prompt as shown below:

Summarization Prompt: Review the six reasoning paths, provide a final binary prediction ("Yes" or "No") along with a summary of your overall assessment.

Reasoning Path 1:

Reasoning Path 2:

…

The output is plain text and is parsed using the same approach as in the proposed method.

3. A self-refine approach that iteratively generates, reviews, and refines responses, allowing the model to detect errors, enhance clarity, and improve accuracy through self-feedback.

A self-refine process is a multi-round conversation between LLM calls. We conduct 6 rounds of refinery, and the prompts of each round are defined as follows.

Base Prompt: The following is a summary of a patient's signs and symptoms extracted from their longitudinal electronic health records. Your task is to predict whether the patient is likely to develop Alzheimer's disease (AD) based on this information. Begin by generating an initial reasoning and prediction. Then, iteratively refine your reasoning and update your prediction across 6 rounds, improving clarity, consistency, and medical logic each time.

Patient Signs and Symptoms:

Patient information: {*patient information*}

For each round, the prompt consists of the following structure:

Round X prompt:

{Based Prompt}

{conversation history (except the 1st round)}

{instruction to refine the answer}

{output instruction}

Round 1 Prompt:

{Base Prompt}

– Initial Reasoning and Prediction:

Reasoning:

Prediction: Yes/No

Round 2 Prompt:

{Base Prompt}

Round 1: {Round 1 Output}

Refine Round 1: Reflect on the previous reasoning. Identify any gaps, oversights, or improvements, and revise accordingly.

Reasoning:

Prediction: Yes/No

Round 3 Prompt:

{Base Prompt}

Round 1: {Round 1 Output}

Round 2: {Round 2 Output}

Refine Round 2:

Analyze the prior reasoning again. Add or revise evidence-based insights if needed.

Reasoning:

Prediction: Yes/No

Round 4 –

{Base Prompt}

{history}

Refine Round 3:

Continue improving accuracy and clarity. Consider any subtle or overlooked signs.

Reasoning:

Prediction: Yes/No

Round 5 –

{Base Prompt}

{history}

Refine Round 4:

Strengthen the reasoning by enhancing coherence and clinical alignment.

Reasoning:

Prediction: Yes/No

Round 6 –

{Base Prompt}

{history}

Final Refinement:

Provide the final and most refined reasoning. This should be your clearest, most confident evaluation.

Reasoning:

Prediction: Yes/No

4. AutoGen Multi-Agent, A multi-agent conversational approach using the AutoGen framework, where domain-specialized agents engage in collaborative dialogue to evaluate AD risk.

Autogen setup is complex. We need to set the system messages for each agent. We also need to define the communications between agents explicitly.

This system simulates a collaborative clinical assessment involving five specialty doctor agents and one AD specialist agent. Each doctor agent independently evaluates the patient's symptoms from a domain-specific perspective and shares their assessment in a multi-turn conversation. The AD specialist agent oversees the discussion, integrates the insights, and provides a final prediction. Agents respond in no fixed order, and performance is evaluated after 1, 2, and 3 rounds of interaction.

Prompt for all agents:

You are a {agent name and role}, participating in a collaborative assessment to determine whether the patient may develop Alzheimer's disease (AD). Review the patient's extracted signs and symptoms and contribute to the conversation by:

• Providing your domain-specific evaluation.

• Responding to others' opinions with constructive feedback.

• Revising your reasoning across rounds based on group input.

{agent-specific prompt}

Patient Signs and Symptoms:

Patient information: {*patient information*}

For the sake of fair comparison and implementation simplicity, the {agent name and role} is kept the same as in our proposed CARE-AD approach, as detailed in Supplementary Note 4.

The {agent-specific prompt} is the same as the instruction {Instruct the agent to assess the patient's situation and identify concerns relating to AD from the agent's domain.}, along with the additional instructions: {specialist-specific guidance}, as detailed in Supplementary Note 4.

Example Conversation Format:

Round 1:

Primary Care Doctor:

Assessment: …

Prediction (Yes/No): …

Neurologist:

Assessment: …

Prediction (Yes/No): …

Geriatrician:

Assessment: …

Prediction (Yes/No): …

Psychiatrist:

Assessment: …

Prediction (Yes/No): …

Clinical Psychologist:

Assessment: …

Prediction (Yes/No): …

AD Specialist (Supervisor Agent):

Summary of Round 1: …

Interim Integration / Concerns Raised: …

Round 2:

Agents respond to each other’s assessments, revise reasoning.

…

Final Round or Consensus:

Agents respond to each other’s assessments, revise reasoning.

AD Specialist:

Final Prediction (Yes/No): …

Justification: …

Summary of Agent Agreement or Key Differing Views: …

Supplementary Note 8: Random forest model implementation details

A random forest (RF) algorithm consists of many decision trees. The "forest" generated by the RF algorithm is trained through bagging or bootstrap aggregating, which helps improve the robustness of the models.

The prediction is made by taking the mean of the output from various trees.

RF models were trained using the scikit-learn package, with a balanced class weight parameter. Hyper-parameters were tuned (grid search) on the 10% model tuning set to determine the optimal values for n_estimators, max_depth (3, 5, 7, 9), bootstrap (true, false), min_samples_leaf (1, 2, 4), min_samples_split (2, 5, 10) and max_features (sqrt, auto). The number of estimators and the maximum depth were adjusted to balance performance and overfitting.

Additionally, a subset of features (max_features) was used for each tree to mitigate the impact of high feature correlation.

TF-IDF calculation

Structured EHR features—including ICD diagnosis codes, medications, and abnormal lab measurements recorded prior to the index date—were processed using term frequency–inverse document frequency (TF-IDF) representations. Each structured code (e.g., an ICD diagnosis or drug class) was treated as a feature. The term frequency (TF) reflects how often a specific code k_i appears in a patient p 's record, while the inverse document frequency (IDF) captures how common that code is across the entire patient population. The TF-IDF weight w_i for each code k_i is calculated as:

$$w_i = tf_{ip} * \log\left(\frac{N}{df_i}\right) \qquad (1)$$

where tf_{ip} is the frequency of code k_i in patient p's records, N is the total number of patients in the training set, and df_i is the number of patients with code k_i in their records. This weighting scheme emphasizes informative and discriminative features while down-weighting codes that are overly common, helping the model better differentiate between AD cases and controls.
